# Supplementary material for: Nitric Oxide-Releasing Thixotropic Hydrogels as Antibacterial and Hemocompatible Catheter Locks
Source: ACS Biomater Sci Eng. 2025 Dec 3;12(1):655–67. doi: 10.1021/acsbiomaterials.5c01661 (PMC12801191; doi:10.1021/acsbiomaterials.5c01661)
Supplement: Supplementary file 1 [file ab5c01661_si_001.pdf]

Supplementary Information

Nitric Oxide-Releasing Thixotropic Hydrogels as Antibacterial and Hemocompatible Catheter Locks

Wuwei Li[1], Loren Liebrecht[2], Surendra Poudel[3], Rebecca Goodhart[1], Sayaji More[1], Jade Montano[2], Derek Lust[2], Qingguo Xu[3], Martin Mangino[2], Xuewei Wang[1*]

[1] Department of Chemistry, Virginia Commonwealth University, Richmond, Virginia 23284, United States

[2] Department of Surgery, Virginia Commonwealth University, Richmond, Virginia 23223, United States

[3] Department of Pharmaceutics, Virginia Commonwealth University, Richmond, Virginia 23298, United States

Email: wangx11@vcu.edu

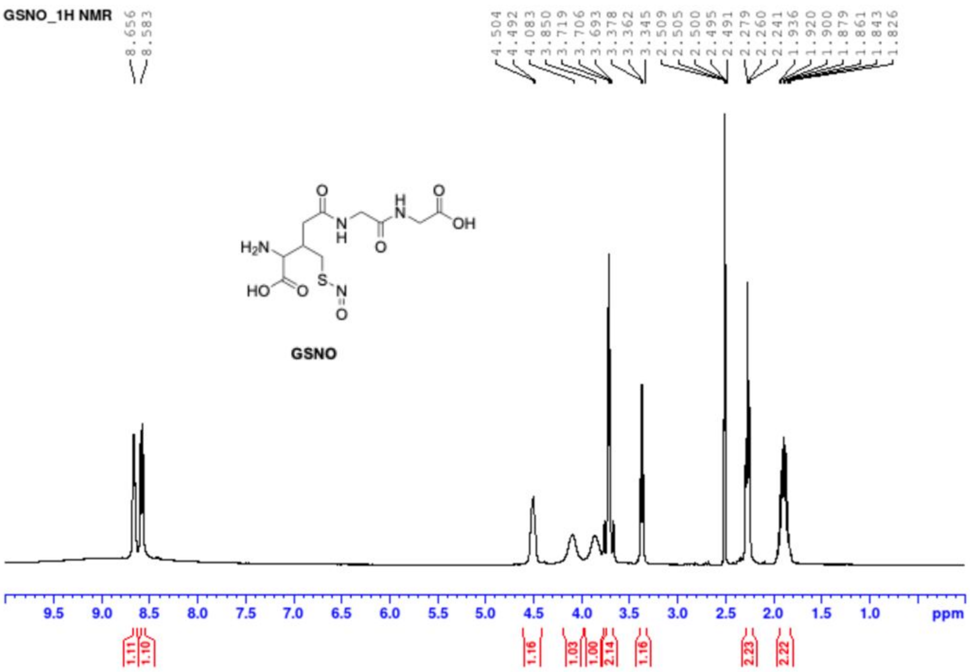
GSNO_1H NMR
8.656
8.583
4.504
4.492
4.083
3.850
3.719
3.706
3.693
3.378
3.362
3.345
2.509
2.505
2.500
2.495
2.491
2.279
2.260
2.241
1.936
1.920
1.900
1.879
1.861
1.843
1.826
GSNO
9.5 9.0 8.5 8.0 7.5 7.0 6.5 6.0 5.5 5.0 4.5 4.0 3.5 3.0 2.5 2.0 1.5 1.0 ppm
1.11 1.10 1.16 1.03 1.00 2.14 1.16 2.23 2.22

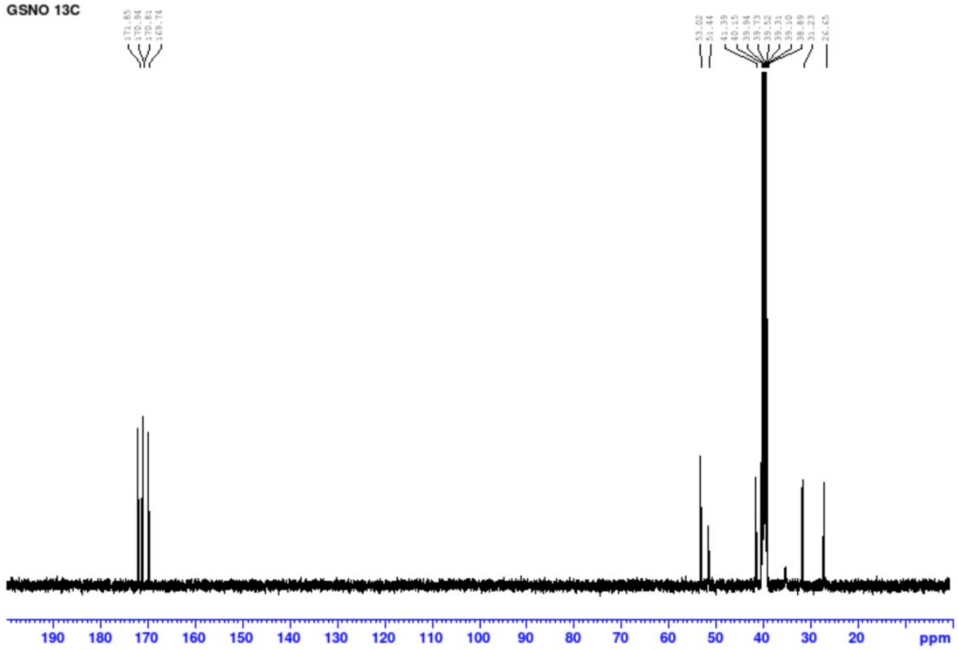
GSNO 13C
190 180 170 160 150 140 130 120 110 100 90 80 70 60 50 40 30 20 ppm

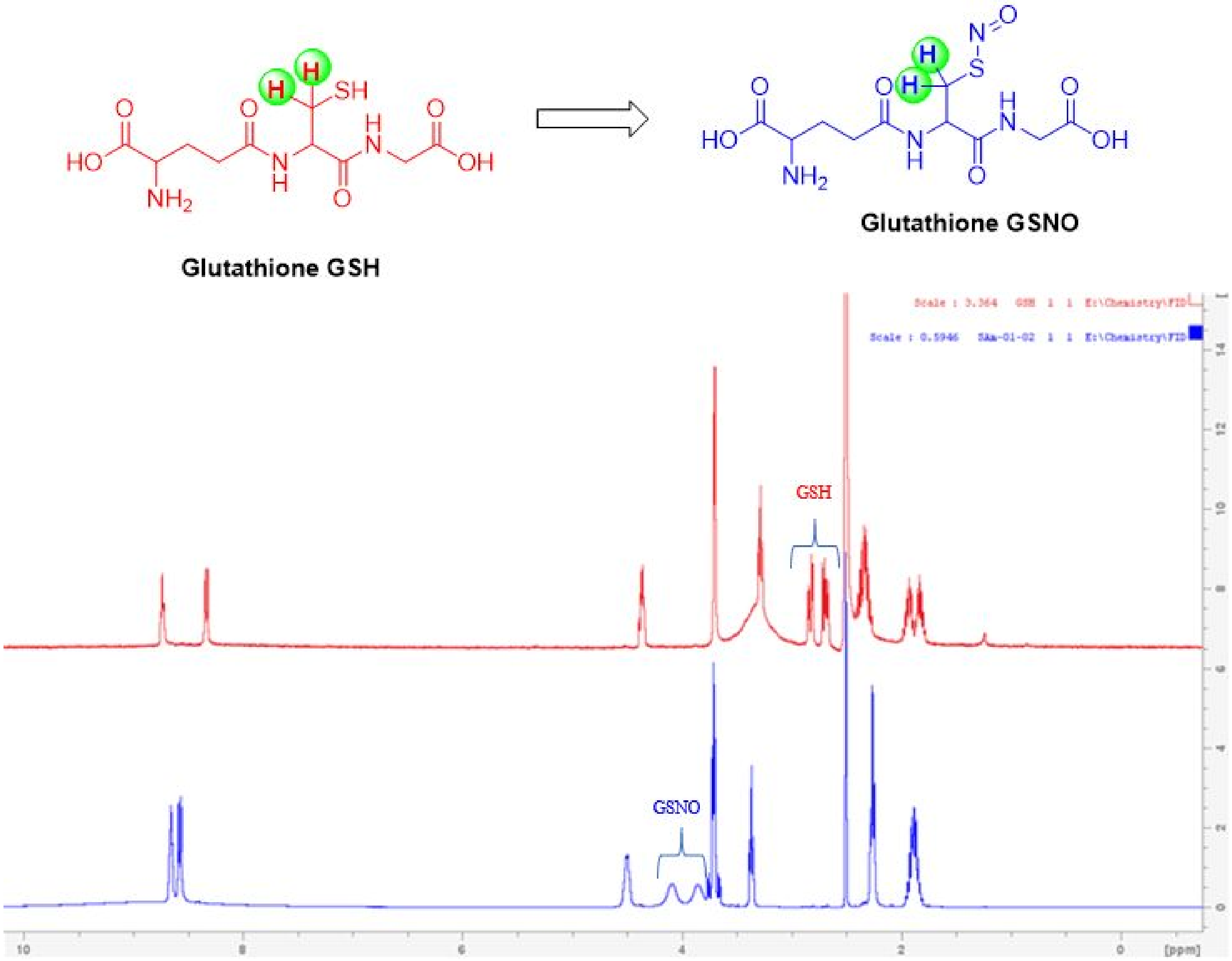

Figure S1. ^{1}H NMR spectrum and ^{13}C NMR spectrum of GSNO.
^{1}H NMR (400 MHz, DMSO): δ 8.65 (bs, 1H), 8.57 (bs, 1H), 4.50 (d, J = 5Hz, 1H), 4.15 ~ 4.0 (m, 1H), 3.92 ~ 3.80 (m, 1H), 3.71 ~ 3.69 (m, 2H), 3.36 (t, J = 6.35, 12.7 Hz, 1H), 2.25 (t, J = 7.44, 14.94 Hz, 2H), 1.93-1.82 (m, 2H).
^{13}C NMR (100 MHz, DMSO): δ; 171.8, 170.9, 170.8, 169.7, 53.0, 51.4, 41.3, 39.9, 31.2, 26.6. The comparison of the ^{1}H NMR spectra of GSH and GSNO is provided to demonstrate the high purity of the GSNO.

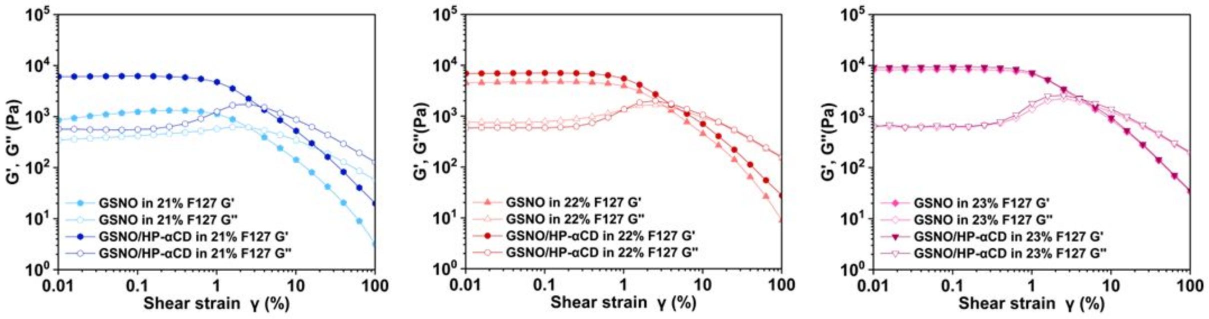

Figure S2. Storage modulus (G′) and loss modulus (G'') of F127 hydrogels as a function of the strain amplitude at a frequency of 10 rad/s at 37 °C.

Table S1. Loss factor (tan δ) of various F127-based hydrogel formulations measured in amplitude sweep tests

Shear strain γ %	21%F127		22% F127		
	GSNO	GSNO/HP-αCD	/	GSNO	GSNO/HP-αCD
**0.0101**	0.403	0.094	0.099	0.171	0.085
**0.0159**	0.387	0.093	0.092	0.167	0.086
**0.0252**	0.367	0.09	0.089	0.161	0.086
**0.04**	0.353	0.09	0.091	0.158	0.085
**0.0634**	0.344	0.088	0.088	0.162	0.085
**0.101**	0.341	0.09	0.09	0.165	0.084
**0.159**	0.343	0.094	0.092	0.171	0.086
**0.252**	0.352	0.105	0.097	0.185	0.09
**0.4**	0.373	0.123	0.13	0.212	0.108
**0.634**	0.414	0.167	0.154	0.255	0.148
**1.01**	0.51	0.265	0.261	0.342	0.248
**1.59**	0.709	0.471	0.481	0.522	0.446
**2.53**	***1.001***	0.779	0.769	0.798	0.744
**4**	1.363	***1.119***	***1.022***	***1.142***	***1.043***
**6.34**	1.823	1.374	1.229	1.572	1.229
**10.1**	2.437	1.66	1.547	2.11	1.498
**15.9**	3.267	2.085	1.969	2.774	1.911
**25.2**	4.453	2.688	2.54	3.7	2.468
**40**	6.286	3.567	3.418	5.398	3.265
**63.4**	9.628	4.786	4.637	8.651	4.371
**101**	17.841	6.474	6.125	16.743	5.732

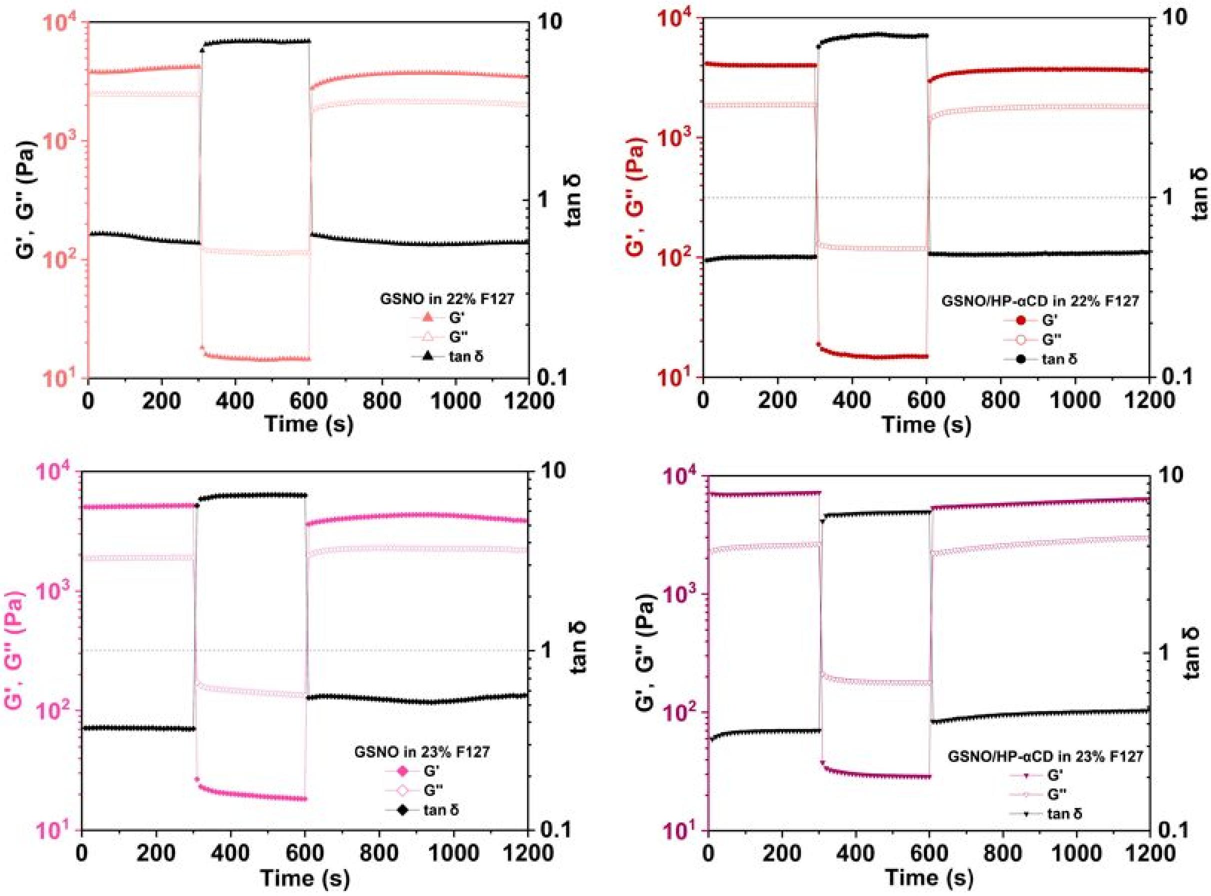

Figure S3. Storage modulus (G′), loss modulus (G''), and loss factor (tan δ) of various F127 hydrogels as a function of time during successive step strain measurements at low (1%), high (100%), and low (1%) shear strains at 37°C.

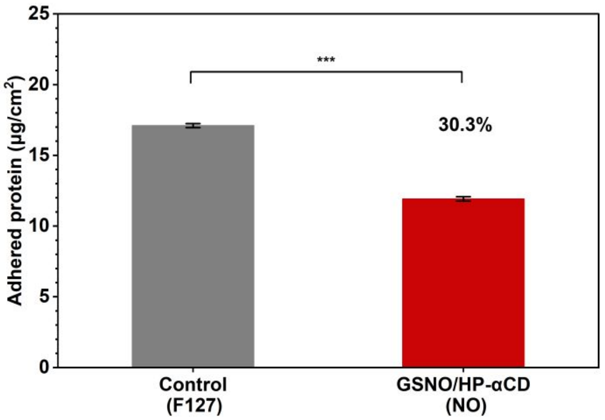

Figure S4. Quantification of proteins adhered to the outer surface of catheter tubes following 24-h exposure to fetal bovine serum at 37°C. n=3; ***$p < 0.001$.

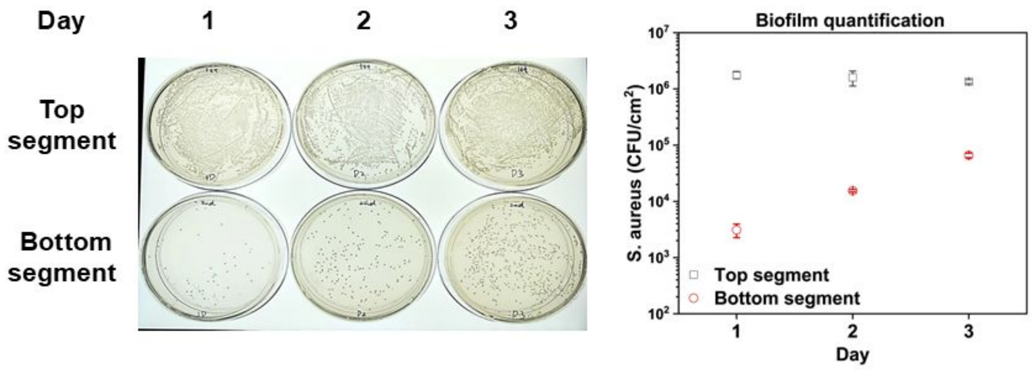

Figure S5. Quantification of *S. aureus* biofilm in the top and bottom segments of the catheter filled with a drug-free F127 hydrogel (n=3).

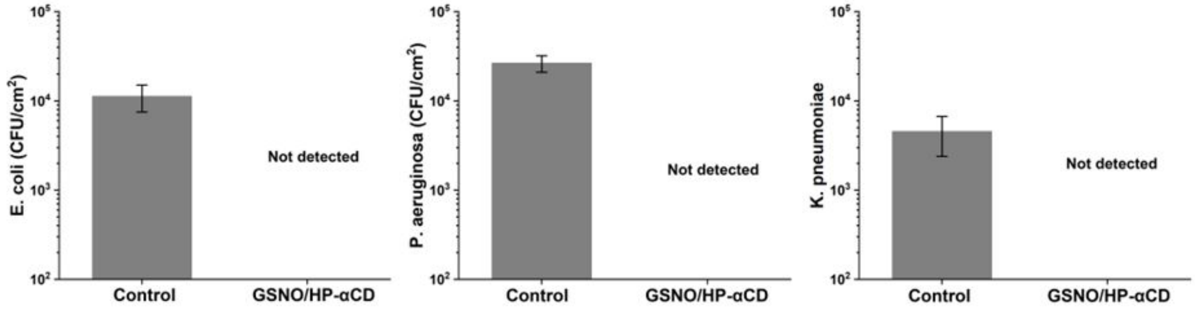

Figure S6. Three-day biofilm tests on the intraluminal surface of the catheter filled with a NO-releasing F127 hydrogel and a control hydrogel (n=3). The limit of detection is 10^2 CFU/cm^2.
